# Olive Oil, Palm Oil, and Hybrid Palm Oil Distinctly Modulate Liver Transcriptome and Induce NAFLD in Mice Fed a High-Fat Diet

**DOI:** 10.3390/ijms20010008

**Published:** 2018-12-20

**Authors:** Rafael C. Sales, Priscylla C. Medeiros, Flavia Spreafico, Patrícia C. de Velasco, Fernanda K. A. Gonçalves, Roberto Martín-Hernández, Diana C. Mantilla-Escalante, Judit Gil-Zamorano, Wilza A. F. Peres, Sergio A. L. de Souza, Alberto Dávalos, Maria G. Tavares do Carmo

**Affiliations:** 1 Instituto de Nutrição Josué de Castro, Universidade Federal do Rio de Janeiro (UFRJ), Rio de Janeiro 21941-902, Brazil; flaviasfer@gmail.com (F.S.); patriciac.velasco@gmail.com (P.C.d.V.); fernandakgst@gmail.com (F.K.A.G.); wilza@nutricao.ufrj.br (W.A.F.P.); 2 Faculdade de Medicina, Universidade Federal do Rio de Janeiro (UFRJ), Rio de Janeiro 21044-020, Brazil; pri.biomed3@gmail.com (P.C.M.); sergioalsouza@gmail.com (S.A.L.d.S.); 3 Laboratory of Epigenetics of Lipid Metabolism, Madrid Institute for Advanced Studies (IMDEA)-Food, CEI UAM+CSIC, 28049 Madrid, Spain; diana.mantilla@imdea.org (D.C.M.-E.); judit.gil@imdea.org (J.G.-Z.); alberto.davalos@imdea.org (A.D.); 4 GENYAL Platform on Nutrition and Health, Madrid Institute for Advanced Studies (IMDEA)-Food, CEI UAM+CSIC, 28049 Madrid, Spain; roberto.martin@imdea.org

**Keywords:** NAFLD, NASH, olive oil, palm oil, hybrid palm oil, high-fat diet, transcriptomics

## Abstract

Nonalcoholic fatty liver disease (NAFLD) is highly prevalent worldwide. The most severe form is nonalcoholic steatohepatitis (NASH). Among risk factors for the development of NAFLD is excessive lipid intake. Since palm (P) oil is the most consumed oil in the world, we aimed to investigate the effects of high-fat diets made with P oil, hybrid palm (HP) oil, or olive (O) oil in liver. Twenty-four male mice (C57Bl/6J) were fed a high-fat diet (41% fat) containing P, HP, or O oils for 8 weeks and compared to a control (C) group fed a chow diet. Adiposity was measured with computed tomography. Body, adipose tissue, and liver weights, as well as liver fat (Bligh–Dyer), blood lipid profile, glucose, and liver enzymes were measured. Liver histology (hematoxylin–eosin) and transcriptome (microarray-based) were performed. ANOVA tests with Newman–Keuls were used. Body weight was increased in the P group (*p* < 0.001) and body fat in the O group (C vs. O *p* ≤ 0.01, P vs. O *p* ≤ 0.05, HP vs. O *p* ≤ 0.05). All high-fat diets disturbed the blood lipid profile and glucose, with marked effects of HP on very low-density lipoprotein cholesterol (VLDL), triglycerides, and alkaline phosphatase (*p* ≤ 0.001). HP had the highest liver fat (42.76 ± 1.58), followed by P (33.94 ± 1.13). O had a fat amount comparable to C (16.46 ± 0.34, 14.71 ± 0.70, respectively). P and HP oils induced hepatocyte ballooning. Transcriptome alterations of the O group were related to amino acid metabolism and fatty acid (FA) metabolism, the P group to calcium ion homeostasis, and HP oil to protein localization. Both P and HP oils induced NASH in mice via disturbed hepatocyte transcription. This raises concerns about the content of these oils in several industrialized foods.

## 1. Introduction

Nonalcoholic fatty liver disease (NAFLD) is a broad-spectrum disease, which encompasses liver steatosis (nonalcoholic fatty liver—NAFL) to inflammation, often with fibrosis (nonalcoholic steatohepatitis—NASH). NASH has been identified as an important risk factor for the development of cirrhosis and hepatocellular carcinoma [1]. This disease is highly associated with lifestyle, with both obesity and metabolic syndrome playing a central role in the ascending prevalence observed nowadays [2,3]. According to recent data, NAFLD is estimated to affect 25% of adult people worldwide [4]. Although this evidence raises concern about the impact of NAFLD in the world, the data available at the moment cannot explain some of the molecular mechanisms involved in the progress of this disease [5]. Therefore, efforts must be made to elucidate the gaps in the knowledge.

Among the known risk factors for the development of NAFLD, inadequate dietary habits are recognized as pivotal [6]. Excessive lipid intake, specifically, seems to be associated with accumulation of fat in animal liver [7], and also in epidemiologic [8] and human intervention [9] studies. Regarding mice, the use of a high-fat diet is the preferable model, since the phenotype of NAFLD developed by this model resembles human NAFLD most accurately. [7,10] As extensively reviewed by Kakimoto & Kowaltowski (2016) [11], high-fat diets can induce accumulation of fat in the liver (steatosis), even with no change in body weight. Several studies reported that insulin signaling impairment and excess production of reactive oxygen species are the main molecular mechanisms involved in accumulation of fat in liver tissue [7,10,11]. The type of fat is mainly important, as in vitro evidence shows that different kinds of fatty acids (FA) exert distinct effects on hepatocytes [12]. In a recent study, we have shown that subjects with advanced liver fibrosis had higher levels of saturated palmitic and stearic fatty acids, as well as oleic acid and total monounsaturated fatty acids (MUFA) in erythrocytes [13]. In fact, FAs are, today, described not only as energy sources, but also as important signaling mediators in several metabolic pathways [14]. In hepatocytes, FAs modulate gene expression through the activation of peroxisome proliferator-activated receptors (PPAR), sterol-regulatory element binding protein 1 (SREBP-1), and toll-like receptors (TLR), among others [15]. In vivo and human studies regarding the effect of fats and oils in the development of NAFLD, however, are scarce. Most studies in animal models focus on the outcomes of lard diet consumption [16], an animal source of saturated fatty acids [17], albeit the most consumed lipid in the world in recent years is palm (P) oil [18].

P oil is extracted from the mesocarp of the fruit derived from the palm tree, *Elaeis guineensis*. The oil is rich in saturated fatty acids, especially palmitic acid—unlike most of the vegetable oils, which are rich in unsaturated fatty acids [19]. For this reason, P oil is semisolid at room temperature, making it suitable for the formulation of ultra-processed food, such as ice cream, cakes, and shortenings [20]. In order to improve the resistance to plagues and the production per hectare, researchers crossbred *Elaeis guineensis* with *Elaeis oleifera*, a variation of the palm tree, developing a new type of palm capable of generating an oil with the same characteristics of P oil, but with superior yield: the hybrid palm (HP) oil [21].

Some authors suggested that, because of the positive effects of HP oil supplementation in blood lipids and antioxidant capacity in humans, and also due to its higher content of oleic acid (a MUFA) and decreased content of palmitic acid, compared to P oil, it should be called “the tropical equivalent of olive oil” [22,23]. Evidence to support this is scarce, however. Concerning the liver, recent data have shown that marmosets fed a high-fat diet containing P or HP oils developed NAFLD, and animals fed a HP diet demonstrated an even higher level of damage to the liver when compared to the group that consumed P [24].

In this scenario, it is critical to investigate the effects of P and HP oils as possible triggers to the development of NAFLD, since the consumption of ultra-processed foods—rich in both oils—is increasing worldwide [25]. It is vital, also, to compare the effects of HP with olive (O) oil. The aim of the present study is to investigate the effects of high-fat diets containing P, HP, and O oils in the liver and the genetic modulation that these oils perpetrate as a mechanism to develop NAFLD.

## 2. Results

### 2.1. Body Weight and Adipose Tissue

**Figure 1:**
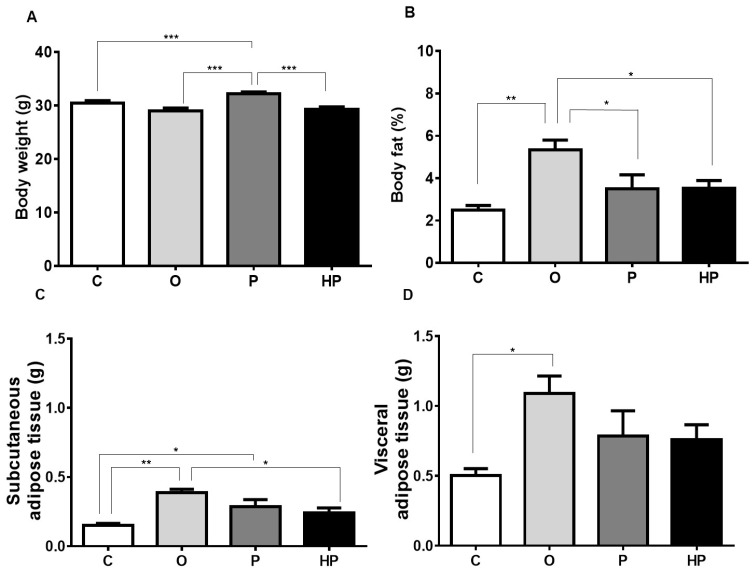
Impact of experimental diets on animals’ body weight (**A**), body fat (**B**), subcutaneous adipose tissue (**C**), and visceral adipose tissue (**D**). Data expressed as mean ± standard error of the mean (*n* = 6). * *p* ≤ 0.05; ** *p* ≤ 0.01; *** *p* ≤ 0.001. C = control group; O = olive oil group; P = palm oil group; HP = hybrid palm oil group.

To assess the dietary impact on body composition, body weight and body fat were measured after 8 weeks of feeding. The P group showed increased body weight compared to the control group (C), HP, and O (*p* ≤ 0.001), as shown in Figure 1A. However, when body fat was assessed, surprisingly the O group demonstrated higher fat content in relation to the three groups (C vs. O *p* ≤ 0.01, P vs. O *p* ≤ 0.05, HP vs. O *p* ≤ 0.05), as shown in Figure 1B. After an adjustment in the analyses, separating adipose tissue compartments, the O group showed substantially more subcutaneous fat than all others (C vs. O *p* ≤ 0.01, P vs. O *p* ≤ 0.05, HP vs. O *p* ≤ 0.05), as shown in Figure 1C, as well as increased visceral fat compared to the C group (*p* ≤ 0.05), as shown in Figure 1D.

### 2.2. Adiposity Measurement by Computed Tomography (CT) in Mice

**Figure 2:**
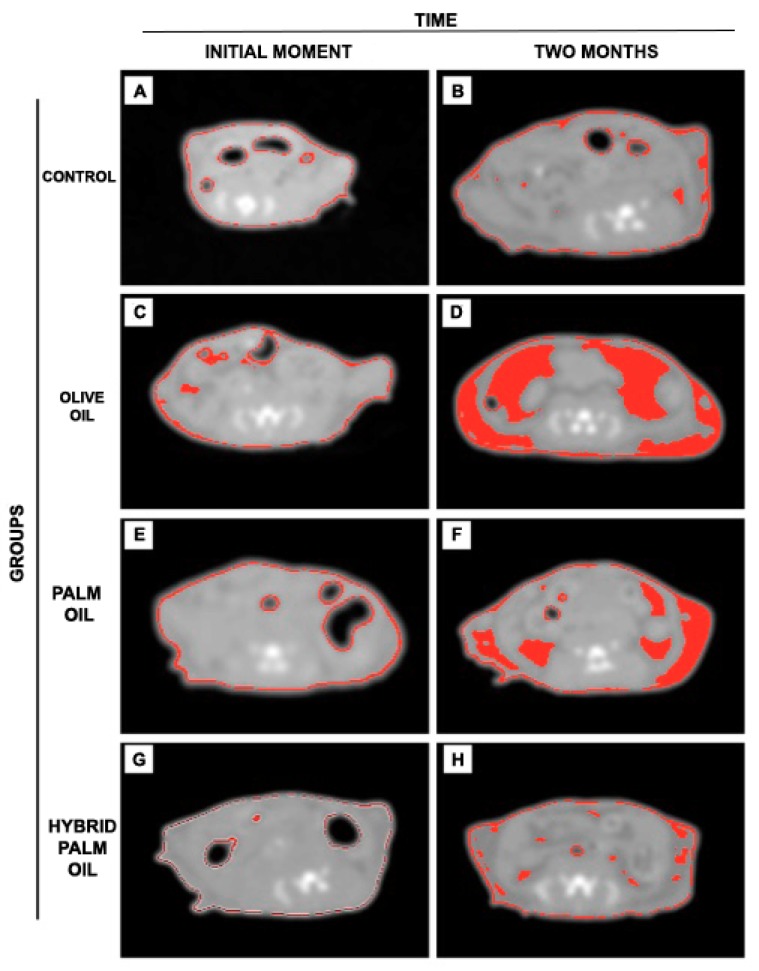
Axial tomographic slices between L4/L5 per dietary group, window levels (WL) -173 HU (Hounsfield units) to -109 HU. Segmentation of body fat (region in red) by ImageJ on the scale referring to adipose tissue of the animals. (**A**) control group (initial moment); (**B**) control group (2 months of diet); (**C**) olive oil group (initial moment); (**D**) olive oil group (2 months of diet); (**E**) palm oil group (initial moment); (**F**) palm oil group (2 months of diet); (**G**) hybrid palm oil group (initial moment); (**H**) hybrid palm oil group (2 months of diet).

To further investigate adipose tissue deposits from baseline to completion of the 8-week study, we applied a CT scan and ImageJ 1.51u software to segment adipose tissue in the tomographic images, which made it possible to follow up groups throughout the study. The O oil group showed greater accumulation of adipose tissue after two months of diet, as shown in Figure 2D, followed by the P group, as shown in Figure 2F, and the HP group, as shown in Figure 2H. The C group showed no significant gain of body fat in two months, as shown in Figure 2B.

### 2.3. Biochemical and Liver Analyses

**Figure 3:**
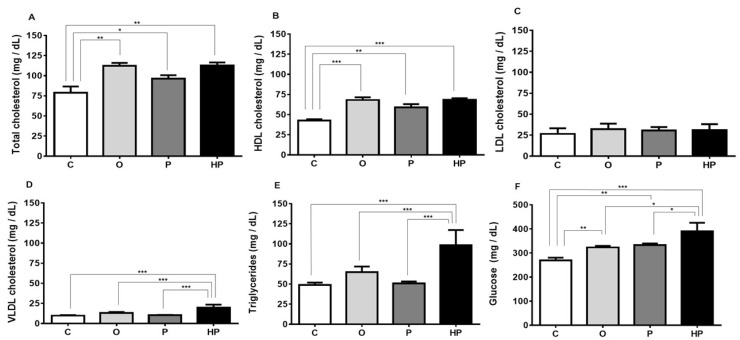
Changes in serum parameters according to each diet. Total cholesterol (**A**); high-density lipoprotein (HDL) cholesterol (**B**); low-density lipoprotein (LDL) cholesterol (**C**); very low-density lipoprotein (VLDL) cholesterol (**D**); triglycerides (**E**); glucose (**F**). Data expressed as mean ± standard error of the mean (*n* = 6). * p ≤ 0.05; ** p ≤ 0.01; *** p ≤ 0.001. C = control group; O = olive oil group; P = palm oil group; HP = hybrid palm oil group.

Regarding serum metabolic parameters, the three high-fat diets increased total cholesterol compared to the C group, with both the O and HP groups demonstrating the highest values (C vs. O *p* ≤ 0.01, C vs. P *p* ≤ 0.05, C vs. HP *p* ≤ 0.01), as shown in Figure 3A. This increase in total cholesterol may be explained by the rise in serum high-density lipoprotein (HDL) cholesterol (C vs. O *p* ≤ 0.001, C vs. P *p* ≤ 0.01, C vs. HP *p* ≤ 0.001), as shown in Figure 3B, promoted by all high-fat diets, whilst no change was observed in serum low-density lipoprotein (LDL) cholesterol concentrations, as shown in Figure 3C. Very low-density lipoprotein (VLDL) cholesterol, on the other hand, behaved differently, with higher concentrations being observed only in the HP group compared to others (*p* ≤ 0.001), as shown in Figure 3D, as well as triglyceride concentration (*p* ≤ 0.001), as shown in Figure 3E. Nevertheless, glucose levels were markedly affected by diets. Comparing to the control, all high-fat diets promoted an increase in serum glucose concentration, with the O and P groups behaving similarly (C vs. O *p* ≤ 0.01, C vs. P *p* ≤ 0.01), as shown in Figure 3F, while the HP group showed the highest concentration (C vs. HP *p* ≤ 0.001, O vs. HP *p* ≤ 0.05, P vs. HP *p* ≤ 0.05), as shown in Figure 3F, suggesting a possible correlation between higher VLDL, triglycerides, and glucose in the HP group.

**Figure 4:**
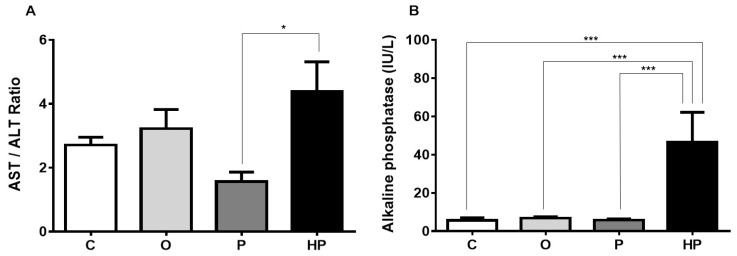

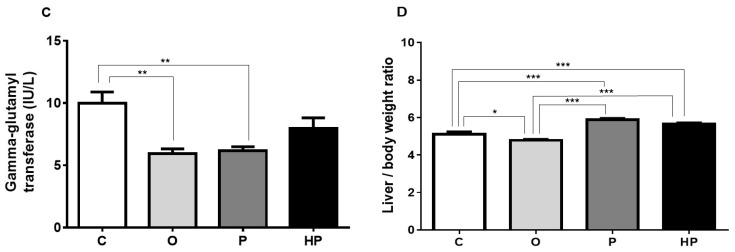
Effects of each diet on liver dynamics. (**A**), AST/ALT ratio; (**B**), alkaline phosphatase; (**C**), gamma-glutamyl transferase; (**D**), Liver weight/body weight ratio. Data presented as mean ± standard error of the mean (*n* = 6). * p ≤ 0.05; ** p ≤ 0.01; *** p ≤ 0.001. AST = aspartate aminotransferase; ALT = alanine aminotransferase; C = control group; O = olive oil group; P = palm oil group; HP = hybrid palm oil group.

Serum liver biomarkers, shown in Figure 4, revealed that the HP diet promoted an increase in the aspartate aminotransferase/alanine aminotransferase ratio (AST/ALT) in relation to the P diet (P vs. HP *p* ≤ 0.05), as shown in Figure 4A. Concerning alkaline phosphatase, a remarkable increase was observed when we compared HP to the other groups (*p* ≤ 0.001), as shown in Figure 4B. Gamma-glutamyl transferase, however, did not differ between HP and the other groups, with the O and P groups exhibiting lower concentrations in relation to the control (C vs. O *p* ≤ 0.01, C vs. P *p* ≤ 0.01), as shown in Figure 4C. We also measured total liver weight and performed a comparison of liver weight/body weight ratio between groups, in order to investigate whether diets promote proportionally an increase in this indicator. The results evidenced that P and HP diets significantly increased this ratio when compared to both the C and O groups (*p* ≤ 0.001), as shown in Figure 4D, whereas the O group unexpectedly showed a lower ratio compared to the C group (*p* ≤ 0.001), as shown in Figure 4D, suggesting that the O diet did not promote an increase in liver size and weight.

Based on these data, we also investigated whether these changes in serum biomarkers and liver weight were due to accumulation of fat in liver tissue. For this reason, we performed a Bligh–Dyer assay to quantify fat concentration in liver tissue of all animals, as shown in Table 1. The analyses revealed that the P and HP groups had substantially higher levels of liver fat (33.94 ± 1.13 and 42.76 ± 1.58, respectively), with great statistical difference among groups (*p* ≤ 0.0001). The higher levels of liver fat in the HP group could be indicative that this diet-induced fat accumulation in the liver potentially caused liver damage, thus resulting in elevation of liver enzymes, as demonstrated in Figure 4.

### 2.4. Histological Analysis

**Figure 5:**
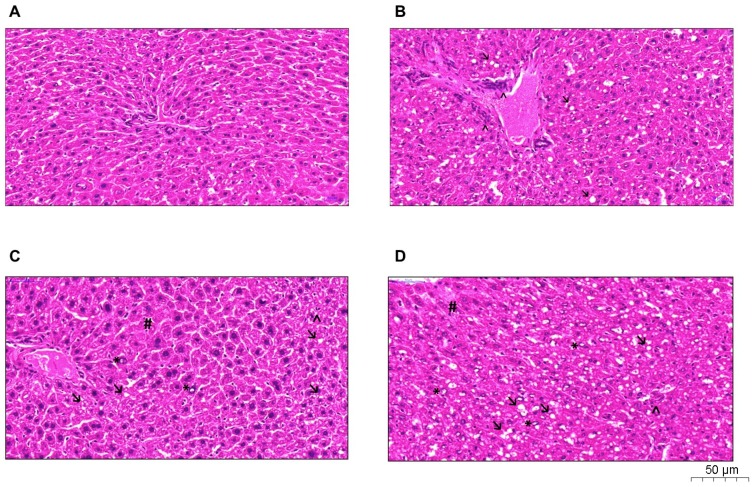
Histological analyses with a hematoxylin-eosin stain of the liver revealed damage induced by high-fat diets, according to the lipid offered: (**A**), control group; (**B**), olive oil group; (**C**), palm oil group; (**D**), hybrid palm oil group. Arrow: lipid vacuoles; Arrowhead: inflammatory infiltrate; Asterisk: nuclear vacuolization; Hash symbol: hydropic degeneration.

The P, HP, and O groups showed higher amounts of lipid vacuoles. Quantification of inflammatory infiltrate, neovascularization, and nuclear vacuolization was observed in greater amounts in the HP group, as shown in Figure 5. In addition, the P and HP groups showed a possible hydropic degeneration (ballooning), characterized by the accumulation of water in the intracellular environment due to the loss of the cell’s ability to maintain ionic balance and fluid homeostasis. NAFLD activity score (NAS) was performed in all groups, as shown in Table 2, and revealed that the O group histology resembles non-NASH NAFLD (score 3), whereas the P and HP groups showed a NASH pattern (score 5 for both).

### 2.5. Transcriptomic Analyses

**Figure 6:**
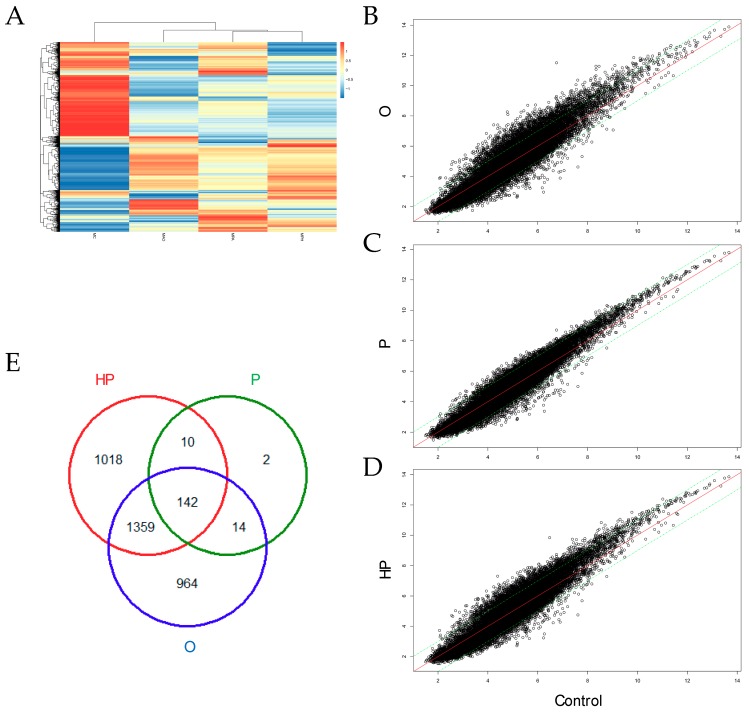
Liver gene expression analysis. (**A**) Heatmap of microarrays data of different dietary groups. Scatter plots in mice liver receiving either olive oil (**B**), palm oil (**C**), or hybrid palm oil (**D**). (**E**) Venn diagram showing the intersections of differentially expressed genes. O, olive oil group; P, palm oil group; HP, hybrid palm oil group.

To determine whether the above-mentioned changes in biochemical parameters modify liver gene expression, we next analyzed the transcriptome of liver mice consuming different types of diets. The whole transcriptome was performed using microarrays gene expression, as shown in Figure 6. Cluster analysis showed that diets containing high-fat differed from the control, as shown in Figure 6A, while the P and HP groups were more similar in gene expression than the O group. Mice consuming O showed a large amount (2479) of differentially expressed genes (DEG), from which 1781 were up-regulated and 698 down-regulated. By contrast, the P group showed the lowest amount of liver transcript changes (168), from which 62 were up-regulated and 106 down-regulated. We also found that the HP group had the highest number (2529) of DEG, of which 1817 up-regulated and 712 were down-regulated. Some of the DEG were common in two or three groups, as shown in Figure 6E. Common transcripts between the O and P groups include adgrb3, nrp2, olfr1222, cspr2, slxl1, dppa2, spin2d, vmn1r114, vmn1r158, and vpreb1, among others. Common transcripts between the P and HP groups include acot9, mrpl32, baiap2, casq2, rpp25, dis3l2, fastkd1, foxo1, irgm1, and lsm1. Common genes among the O and P groups include more than 1300 genes.

**Figure 7:**
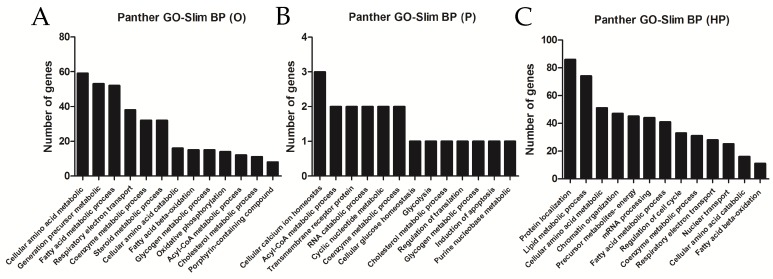
Functional enrichment analysis of differentially expressed genes. Panther Gene Ontology (GO) biological processes (BP) of over-represented terms of liver transcriptome according to the lipid supplementation: (**A**): O, olive oil group; (**B**): P, palm oil group; (**C**): HP, hybrid palm oil group.

Using databases for functional analysis enrichment of DEG showed that most modulated genes were found to be related to cellular amino acid metabolism, FA metabolic processes, steroid and cholesterol metabolic processes, and the FA beta-oxidation pathway, among others, for the O group, as shown in Figure 7A; cellular calcium ion homeostasis or acyl-CoA metabolic processes, among others, for the P group, as shown in Figure 7B; and protein localization, lipid metabolic processes, FA metabolic processes, and FA acid beta-oxidation, among others, for the HP group, as shown in Figure 7C. Suggesting overall that lipid metabolism pathways are dysregulated in liver transcriptome in response to a high-fat diet. 

## 3. Discussion

The food industry has been extensively using lipid sources that could maintain the texture and increase the shelf life of foodstuffs [24]. For many years, these products were primarily made up of partially hydrogenated fats rich in trans FA, in foods such as margarines, cookies, and ice creams. Several studies have demonstrated that the use of trans fat can trigger disturbances in health, such as obesity, cardiovascular diseases, and other factors related to metabolic syndrome. Since then, the industry has been using other lipid sources for the replacement of trans fats in foods [24,25]. P oil currently represents the most used vegetable oil by the food industry, even though little is known about the health risks related to it [18]. In this sense, the results of this study, comparing high-fat diets rich in P or HP oils with O oil, caused in mice distinct biomolecular effects and, therefore, signal important deleterious effects caused by their excessive intake.

High-fat diets are known to increase body weight and adiposity in rodents [26], making this type of diet the main choice for the study of obesity in animal models. More recently, high-fat diets have been used to induce NAFLD in animals, in order to study both the pathophysiology and possible treatments for this disease [27,28]. Indeed, among the animal models of NAFLD, the “high-fat diet induced” model is referred to as the one that mimics human NAFLD the most [28]. In our study, not all animals fed a diet rich in lipids gained weight. The body weight of the groups fed with O or HP oils were similar to the C group, whereas the P group showed a significant increase compared to all the others, suggesting that the type of fat influences how body weight is changed throughout the lifespan. This is in accordance with other studies, reviewed by Hariri & Thibault [26]. Factors like saturation of the FA and size of the carbon chain directly influence the bioavailability of the FA consumed. Saturated FAs (SFAs), for instance, are poorly used for immediate energy upon intake and absorption, so they tend to be stored in adipose tissue. Polyunsaturated fatty acids (PUFA) tend to be immediately oxidized or incorporated on another function. MUFA, otherwise, are controversial, with studies showing conflicting results [26].

Although many studies in humans [29,30] promote O oil (high in MUFA) as an oil with therapeutic use, there is no consensus about the amount needed for the benefits without any harm. Evidence shows an association between MUFA intake and waist-hip ratio in an obese Mediterranean population [31]. On that matter, our study indicated that although the O group weight did not differ from C group, it had increased body fat compared to all groups, measured both directly and via CT. A physiological explanation for that may be inferred from the study of Benner et al. [32], where the authors showed that oleic acid is preferentially incorporated in triglycerides in rabbit hepatocytes, to be exported after to adipose tissue. This mechanism may also partially explain our results in mice regarding the lower percentage of fat found in the liver of the O group, as shown in Table 1, accompanied by greater accumulation of adipose tissue, investigated by CT scan.

In fact, the fat located in the liver measured by the Bligh–Dyer method revealed that the C and O groups did not differ, whilst the P group showed increased liver fat and HP showed the highest liver fat amount. Liver histology analyses confirmed these data, when compared to animals in the C group, the O group showed small amounts of lipid vacuoles in tissue. Both the P and HP groups exhibited signs of hepatocytes ballooning, yet only HP demonstrated significant inflammatory infiltration, neovascularization, and nuclear vacuolization. According to the definition of NAFLD by the American Association for the Study of Liver Diseases [1], NAFLD can be classified in different stages: NAFL or NASH. NAFL (onset of disease) is defined as a simple accumulation of fat in liver tissue with absence of hepatocyte ballooning, while NASH (the most severe form of the disease) is characterized as fat accumulation and inflammation in liver tissue with hepatocyte ballooning, with or without fibrosis—as we observed in the P and HP groups. The NAS score also revealed that the P and HP liver tissues resembled NASH, while the O group presented a more NAFL pattern. The causal relationship of the P and HP diets with the development of NASH, however, is new evidence, and to the best of our knowledge, not previously demonstrated. Thus, our study showed that high-fat P and HP diets act differently in the liver tissue of mice, causing more hepatic dysfunction in the P and HP groups than the O and C groups. Whether these effects may be associated with changes in liver mitochondrial bioenergetics is currently being investigated in our laboratory, as different dietary lipid sources have been shown to impair liver mitochondrial bioenergetics in distinct ways [33].

In another study with marmosets [24], we showed that both P and HP oils altered hepatic metabolism and caused lipid accumulation, with HP performing worst. We also demonstrated in another study that palmitic acid—the main SFA present in P and HP oils—is independently associated with liver fibrosis in patients with NAFLD [13]. In fact, the NOD-like receptor family pyrin containing 3 (NLRP3) inflammasome has been associated with the development of NASH and palmitic acid increases the expression of NLRP3 inflammation and induces IL-1b e TNF—a secretion in liver favoring the progression of NAFLD [34]. With this evidence, we can hypothesize that a high intake of palmitic acid through P or HP oils is somewhat involved in liver damage in mice, inducing NASH in just eight weeks of feeding. Regarding O oil, further studies are necessary to investigate whether mice fed for a longer time can predispose the development of NASH. The results of the present study demonstrated that the type of fat, in the context of a high-fat diet, can predispose mice to NAFLD, with a difference in the phenotype of the disease according to the fat consumed. This is problematic, since NAFLD is a spectrum that, if not treated, can progress to liver cirrhosis and hepatocellular carcinoma [1]. High-fat diets have been shown to be both the promoter and initiator of liver cancer in mice, when animals were fed long-term [35,36].

Since liver orchestrates the metabolism of several biochemical parameters [37], we looked into the effects of the high-fat diets on these aspects. Serum total and HDL cholesterol increased in all experimental groups, with the most prominent enhancement visualized in the O and HP groups, while LDL cholesterol did not present statistical difference between groups. O and HP are oils with a higher content of oleic acid than P oil, a fatty acid for decades known to increase HDL [38]. Both VLDL and triglycerides were elevated only in the HP group, with significant difference compared to the others. In addition, glucose was increased in all groups in relation to the C group, yet HP caused the highest elevation of all, with statistical difference between all groups. This scenario can be interpreted as insulin resistance in different stages, with the worst case being the HP group. As extensively reviewed by Sparks et al. [39], insulin resistance is responsible for VLDL overproduction and hypertriglyceridemia, since insulin is involved in all lipoprotein metabolism and secretion. According to the increase in glucose, VLDL, and triglycerides, we can infer that HP oil caused insulin resistance in mice. To confirm this hypothesis, future works in this field will be necessary.

Other serum biomarkers were also differentially affected by the diets. The AST/ALT ratio was higher in HP groups when compared exclusively with the P group. This index reflects hepatic damage and can predict liver impairment, insulin resistance, and incident metabolic syndrome [40,41,42]. Thus, mice from the HP group appear to face more liver disturbances than the other groups, in accordance with data from marmosets with the same oil [24]. Alkaline phosphatase was also significantly increased in HP mice serum, while P and O mice were comparable to C mice. This enzyme is traditionally related to cholestatic injury [43], yet there is evidence of cases of NAFLD with high alkaline phosphatase [44]. Gamma-glutamyl transferase concentration, on the other hand, was not different between the HP and C groups, but was lower in the O and P groups compared to the C group. Low levels of this enzyme in serum are not common in studies or in clinical practice. According to Ndrepepa and Kastrati (2016) [45], gamma-glutamyl transferase’s most pivotal role is the cleavage of glutathione, liberating the three amino acids that form it (glutamic acid, cysteine, and glycine) and making it possible for the cell to absorb these amino acids to synthetize glutathione again, or to utilize them in other metabolic pathways. Evidence associates high gamma-glutamyl transferase with cardiovascular diseases [45,46], however, neither of them address the issue of the low serum concentration of this enzyme. In the absence of accurate evidence comprising high-fat diets and glutathione to explain this decrease, we can only hypothesize that this effect we observed is somewhat related to glutathione availability. This elicits further investigation concerning liver gamma-glutamyl transferase, glutathione metabolism, and high-fat diets containing either P or O oils.

Previous studies have compared the consumption of palmitic acid vs. oleic acid, both in rodents [47] and humans, under normal level of lipids. While similar effects on lipid level profiles were found in normocholesterolemic men and women, in rodents, certain differences were found in plasma lipids but not in liver tissue cholesterol levels [47]. Few studies have compared the effects of these fats under high-fat diet conditions. In a recent study, comparing O oil vs. P stearin under a high-fat diet, Meidan et al. [48] found that mice receiving either P or O fats showed an increased body weight, elevated blood glucose levels, and a fatty liver phenotype. In rats, high dietary intake of P oil was found to compromise glucose tolerance, while O compromised liver lipid metabolism and integrity [49]. Overall, the above-mentioned studies regarding lipid metabolism suggest the response to dietary lipids might be exacerbated in the context of a high-fat diet. Moreover, physiological conditions (i.e., obesity) might also influence the response to dietary lipids [50].

To the best of our knowledge, our study is the first to compare the liver transcriptome of mice fed high-fat diets containing O, P, and HP oils. Although we do not discard that response to dietary fats might be different in conditions of high-fat from that of a normal diet, our data suggest that the total amount of fat might be a major driver of transcriptomic changes. Previous studies have evaluated the impact of a high-fat diet on the liver transcriptome [51] in rodents and in agreement with our data, most regulated genes were related to FA metabolism. Regarding inflammatory genes, in accordance with previous works [49], we did not observe major changes in classical inflammatory genes in the liver, including IL-6, IL-1β, MCP1, or TNFα [48]. Interestingly, cpt1a, a major player in oxidation of FA [52] was down-regulated in the HP group, while the Scd1 gene was up-regulated in both the P and HP groups. Overall, our data suggest that different oils produce a large amount of changes in the liver transcriptome, particularly for the O and HP oils. Which of these changes, at mRNA levels, are a determinant for the biological effects observed in the whole organism, needs further characterization.

Our results suggest that high-fat diets containing MUFA or SFA disturb fat distribution and lipid metabolism in distinct ways, contributing to NAFLD establishment. This indicates that the total amount of fat consumed is the main driver of liver disease development, and the type of fat can distinctly influence the degree of NAFLD developed. The limitations of our study are that it was performed under a high-fat diet context to better resemble the western diet, however, we cannot predict whether these oils would induce the same metabolic disarrangements if animals were fed a diet with normal fat content. In addition, to understand if these results can be translated to humans, further studies involving NAFLD patients and these oils must be performed. Since P oil is the most cultivated, commercialized, consumed, and utilized oil in the world [18] and HP oil is promoted as a healthy alternative for it [22,23], our data suggest that attention must be given to the liver outcomes observed here in mice before boosting these oils as safe and healthy.

## 4. Materials and Methods 

### 4.1. Animals

Twenty-four male mice (*Mus musculus*), C57Bl/6J, at 8 weeks of age, were used in this study. They were raised and kept at Biotério de Roedores of Instituto de Nutrição Josué de Castro, Universidade Federal do Rio de Janeiro (UFRJ), Brazil, and were housed in groups (three animals per cage). The room was controlled, with 12-h light/12-h dark cycle, average temperature of 23 °C ± 2 and humidity of 50% ± 5. The animals had free access to food and water.

All procedures followed the Guide for the Care and Use of Laboratory Animals. This study was approved by the Ethics Committee for The Use of Animals in Research of Universidade Federal do Rio de Janeiro—UFRJ (protocol number 049/17).

### 4.2. Experimental Dietetic Protocol

The animals were randomly assigned to one of the four groups, with six animals each: control (C) group, fed with a chow diet (Laboratory Rodent Diet, LabDiet^®^); or one of three high-fat diets (41% fat), differing in the lipid sources: olive oil (O) group, containing olive oil; palm oil (P) group, containing palm oil; or hybrid palm oil (HP) group, containing hybrid palm oil. The high-fat diets were formulated by Prag Soluções (São Paulo, Brazil), following the recommendations of the American Institute of Nutrition for rodents. The formulation of the high-fat diets is described in Table 3. Palm oil and hybrid palm oil were provided by Agropalma (Pará, Brazil). Olive oil was purchased from a local market. All groups were fed for 8 weeks.

### 4.3. Body Weight and Computed Tomography

Body mass was measured at the initial day (T0), before the dietary approach, and all across the intervention time. Computed tomography (CT) scans were performed in order to assess adipose tissue and liver images. All animals underwent CT scans at T0 and at the final time (T1), after the diets. The images were taken on Optima PET/CT560 equipment (Milwaukee, WI, USA), located at the Nuclear Medicine Service of the Hospital Universitário Clementino Fraga Filho of Universidade Federal do Rio de Janeiro (Rio de Janeiro, Brazil), and processed on AW4.6 software. The settings for CT scans were supine position, abdomen window levels (WL) of -173 HU to -109 HU (HU scale calculated specifically for the animals in this study), slices of 0.62 mm, 140 Kv, and 320 mAs. Segmentation of adipose tissue was performed by Image J.51u software, where a tomography slice of each animal was used to count the Hounsfield unit (HU)-scale pixels corresponding to the fat tissue of the animals. ImageJ is public domain software that is well used in automatic or manual image processing in studies related to adipose tissue segmentation [53,54,55].

### 4.4. Biological Sample Collection and Liver Histological Analyzes

At T1, after 4 h of fasting, all animals were anesthetized intraperitoneally with 300 mg/kg of ketamine hydrochloride and 30 mg/kg of xylazine hydrochloride, and blood was collected by cardiac puncture. Subsequently, liver and adipose tissue samples were collected. The blood was centrifuged (4000× *g*, 4 °C, 10 min) in order to collect serum samples and sent under refrigeration to Laborlife Clinical Analysis (Rio de Janeiro, Brazil) for biochemical analysis, such as total cholesterol, high-density lipoprotein cholesterol (HDL), low-density lipoprotein cholesterol (LDL), very low-density lipoprotein cholesterol (VLDL), triglycerides, aspartate transaminase (AST), alanine transaminase (ALT), alkaline phosphatase, gamma-glutamyl transferase, and glucose. For these analyses, colorimetric chemical kinetic assays were used with an automated clinical analyzer (Metrolab 2300, Geneva, Switzerland). Liver and adipose tissues were weighted. A liver sample was used for histologic analysis: 100 mg of liver tissue was collected to perform transcriptome analysis, so it was immersed in RNAlater^®^ Stabilization Solution (Thermo Fisher, Waltham, MA, USA), according to the manufacturer’s instructions, for further analysis. Then, it was stored at −80 °C, with the rest of the sample. Tissues (defrosted) were fixed in formalin, paraffin embedded, and sections were stained with hematoxylin and eosin. Hematoxylin and eosin staining was used to evaluate the lesion pattern and whether there was any type of cellular immune response. The analyses were made based on the score system of Kleiner et al. [56]. In summary, the histological slices were scanned using a slide scanner (Pannoramic MIDI - 3DHISTECH, Budapest, Hungary), where the entire liver tissue sample was evaluated. The results were analyzed by an external expert pathologist and were based on observation of the degree of steatosis, focal inflammation, diffuse inflammation, and nuclear vacuolation. NAFLD activity score (NAS) was performed in order to clarify the stage of liver disease in the animals [56].

### 4.5. Liver Fat Assessment

To assess liver fat, 200 mg of liver samples were submitted to lipid extraction according to Bligh and Dyer (1959) [57] and was determined by the gravimetric method. Results are expressed as a percentage of the sample.

### 4.6. Liver Transcriptome

Total mRNA of the liver samples was extracted with a PrepEase^®^ kit (Affymetrix), following the manufacturer’s instructions. mRNA integrity was assessed with NanoDrop-1000^®^ equipment (Thermo Fisher) before microarray-based transcriptomic analyses. Gene expression analysis was performed using Affymetrix Clariom S Mouse Assay microarrays. The R Bioconductor oligo package was used for data processing in conjunction with the pd.clariom.s.mouse annotation package. After background correction and robust multichip average (RMA) normalization, expression data was obtained for a total of 29,129 features and 19 samples. Samples within the same experimental group which correlated poorly were removed. Thus, one sample was removed from C, PH, and O groups, respectively. Then microarray probes were annotated with the corresponding gene symbol, and not available (NA’s) gene symbols were removed from the expression matrix. Differential expression levels between experimental groups were assessed with the R Bioconductor limma package. Genes with a false discovery rate (FDR) lower than 0.05 were considered as statistically significant. Gene Ontology (GO) analysis of biological process (BP) was performed using the Panther Database (http://www.pantherdb.org/pathway/) and using GO slim annotation. Only over-represented (or under-represented) GO annotations with an FDR < 0.05 were considered. 

### 4.7. Statistical Analysis

The software GraphPad Prism 7.0 was used for statistical analysis. An ANOVA test, along with a Newman–Keuls post-hoc test were performed, adopting a significance level of *p* ≤ 0.05. Results are expressed as means ± standard error of the mean.

## 5. Conclusions

The consumption of high-fat diets containing either olive, palm, or hybrid palm oils during eight weeks can predispose mice to the development of NAFLD. Olive oil promoted low liver fat accumulation, but prompted lobular inflammation, with transcriptomic modulation of amino acid and fatty acid metabolism. Palm and hybrid palm oils, in other ways, induced not only liver fat accumulation, but also hepatocyte ballooning and lobular inflammation, resembling the nonalcoholic steatohepatitis pattern. Transcriptome alterations in the palm oil-fed group were related to calcium ion homeostasis, whereas in the hybrid palm oil-fed group were related to protein localization. As demonstrated in the present study, high dietary intake of palm and hybrid palm oils can rapidly induce NASH in mice. These results raise concern about the high content of these oils in the most-consumed processed food products around the world.

## Figures and Tables

**Table 1:** Liver fat measurements in mice submitted to a high-fat diet protocol (%).

Group	Liver Fat (%)	Statistical Significance Versus Control	Statistical Significance Versus Olive oil	Statistical Significance Versus Palm Oil	Statistical Significance Versus Hybrid Palm Oil
Control	14.71 ± 0.70	-	*p* = 0.1189	*p* < 0.0001	*p* < 0.0001
Olive oil	16.46 ± 0.34	*p* = 0.1189	-	*p* < 0.0001	*p* < 0.0001
Palm oil	33.94 ± 1.13	*p* < 0.0001	*p* < 0.0001	-	*p* < 0.0001
Hybrid Palm oil	42.76 ± 1.58	*p* < 0.0001	*p* < 0.0001	*p* < 0.0001	-

Measured by the Bligh–Dyer method; data presented as means ± standard error of the mean (*n* = 6).

**Table 2:** NAFLD (nonalcoholic fatty liver disease) activity score (NAS) based on histological analyses.

Group	Grade of Steatosis	Lobular Inflammation	Ballooning	NAFLD Activity Score
Control	1	0	0	1
Olive oil	1	2	0	3
Palm oil	2	2	1	5
Hybrid Palm oil	2	2	1	5

**Table 3:** Experimental diet composition shown as g/100 g.

Ingredients	g/100 g
Ground corn	8.80
Wheat middlings	8.50
Rice bran	8.00
Sucrose	19.50
Dehulled soybean meal	5.00
Casein	7.00
Powder milk	7.00
Albumin	11.20
Chicken meal	2.90
Soybean oil	2.00
P, HP, or O oils	14.30
Fiber	2.00
Vitamins and minerals mix	3,86
Butylhydroxytoluene	0.02
*Kcal/100 g*	422.08
Protein	16%
Carbohydrate	43%
Lipid	41%

## Abbreviations

NAFLD	Nonalcoholic fatty liver disease
NAFL	Nonalcoholic fatty liver
NASH	Nonalcoholic steatohepatitis
FA	Fatty acids
MUFA	Monounsaturated fatty acids
PPAR	Peroxisome proliferator–activated receptors
SREBP-1	Sterol-regulatory element binding protein 1
TLR	Toll-like receptors
P	Palm
HP	Hybrid palm
O	Olive
CT	Computed tomography
NAS	Nonalcoholic fatty liver disease activity score
AST/ALT	Aspartate aminotransferase/alanine aminotransferase
DEG	Differentially expressed genes
SFA	Saturated fatty acids
NLRP3	NOD-like receptor family pyrin containing 3
T0	Initial day
T1	Final time
HU	Hounsfield unit
HDL	High-density lipoprotein cholesterol
LDL	Low-density lipoprotein cholesterol
VLDL	Very low-density lipoprotein cholesterol
GO	Gene ontology
BP	Biological process
